# Choline and methionine differentially alter methyl carbon metabolism in bovine neonatal hepatocytes

**DOI:** 10.1371/journal.pone.0171080

**Published:** 2017-02-02

**Authors:** Tawny L. Chandler, Heather M. White

**Affiliations:** Department of Dairy Science, University of Wisconsin-Madison, Madison, WI, United States of America; Duke University School of Medicine, UNITED STATES

## Abstract

Intersections in hepatic methyl group metabolism pathways highlights potential competition or compensation of methyl donors. The objective of this experiment was to examine the expression of genes related to methyl group transfer and lipid metabolism in response to increasing concentrations of choline chloride (CC) and DL-methionine (DLM) in primary neonatal hepatocytes that were or were not exposed to fatty acids (FA). Primary hepatocytes isolated from 4 neonatal Holstein calves were maintained as monolayer cultures for 24 h before treatment with CC (61, 128, 2028, and 4528 μmol/L) and DLM (16, 30, 100, 300 μmol/L), with or without a 1 mmol/L FA cocktail in a factorial arrangement. After 24 h of treatment, media was collected for quantification of reactive oxygen species (ROS) and very low-density lipoprotein (VLDL), and cell lysates were collected for quantification of gene expression. No interactions were detected between CC, DLM, or FA. Both CC and DLM decreased the expression of methionine adenosyltransferase 1A *(MAT1A)*. Increasing CC did not alter betaine-homocysteine S-methyltranferase *(BHMT)* but did increase 5-methyltetrahydrofolate-homocysteine methyltransferase *(MTR)* and methylenetetrahydrofolate reductase *(MTHFR)* expression. Increasing DLM decreased expression of *BHMT* and *MTR*, but did not affect *MTHFR*. Expression of both phosphatidylethanolamine N-methyltransferase *(PEMT)* and microsomal triglyceride transfer protein (*MTTP)* were decreased by increasing CC and DLM, while carnitine palmitoyltransferase 1A *(CPT1A)* was unaffected by either. Treatment with FA decreased the expression of *MAT1A*, *MTR*, *MTHFR* and tended to decrease *PEMT* but did not affect *BHMT* and *MTTP*. Treatment with FA increased *CPT1A* expression. Increasing CC increased secretion of VLDL and decreased the accumulation of ROS in media. Within neonatal bovine hepatocytes, choline and methionine differentially regulate methyl carbon pathways and suggest that choline may play a critical role in donating methyl groups to support methionine regeneration. Stimulating VLDL export and decreasing ROS accumulation suggests that increasing CC is hepato-protective.

## Introduction

**Fig 1:**
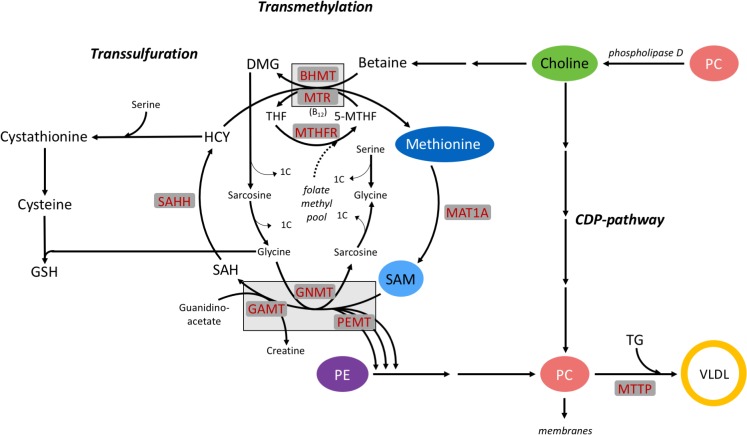
Intersection between pathways of choline and methionine metabolism in the transmethylation cycle and key enzymes that control methyl group transfer: methionine adenosyltransferase 1A (MAT1A), betaine-homocysteine S-methyltranferase (BHMT), 5-methyltetrahydrofolate-homocysteine methyltransferase (MTR), methylenetetrahydrofolate reductase (MTHFR), microsomal triglyceride transfer protein (MTTP), S-adenosylhomocysteine hydrolase (SAHH), glycine-N-methyltransferase (GNMT), guanidinoacetate N-methyltransferase (GAMT), phosphatidylethanolamine N-methyltransferase (PEMT), vitamin B_12_ (B_12_), dimethylglycine (DMG), glutathione (GSH), homocysteine (HCY), S-adenosylmethionine (SAM), S-adenocylhomocysteine (SAH), tetrahydrofolate (THF), phosphatidylethanolamine (PE), phosphatidylcholine (PC), very low-density lipoprotein (VLDL).

Labile methyl groups are required to support hepatic metabolism and are involved in pathways of transmethylation, transsulfuration, the folate cycle, and synthesis of methylated compounds. Methyl donors such as betaine, choline after oxidation to betaine, methionine, and folic acid can supply methyl groups; however, extensive rumen microbial degradation limits hepatic supply of these in functional ruminants [1,2]. Rumen-protection and gastro-intestinal administration of choline and methionine increase arterial concentrations of choline metabolites [3,4] and portal vein concentrations of methionine [5]. Given that metabolism of both choline and methionine provides methyl groups, pathways of their catabolism and endogenous remethylation intersect. Exogenous sources or endogenously remethylated methionine are required for the methylation of S-adenosylmethionine (SAM), the universal biological methyl donor [6] in the transmethylation pathway (Fig 1). Although microbial protein supplies methionine for intestinal absorption in ruminants, this microbial protein has to meet the requirements of methionine as a source of methyl groups and a potential limiting amino acid for net protein synthesis to support ruminant growth and production [7,8]. Methionine supply is further challenged by its use for the *de novo* synthesis of choline when choline is unavailable. In lactating goats, an equivalent of 28% of absorbable methionine is involved in choline synthesis [9]. While choline recycled from phosphatidylcholine (PC) can supply labile methyl groups for the regeneration of methionine (Fig 1) [10], this potential drain of PC may impair lipid membrane integrity and very-low density lipoprotein (VLDL) secretion by the liver, as PC is integral to both [11,12].

Although these interactions most likely confound the requirements of both choline and methionine, they potentially allow for sparing of one by the other. Previous data demonstrated that supplying choline decreased the irreversible loss of methionine in the transmethylation pathway [10], possibly sparing it for anabolic output. Potential sparing between choline and methionine may aid in meeting their biological priorities at distinct physiological states. This is of critical importance during the transition period in dairy cattle, when the onset of lactation increases the demand for methylated compounds to be secreted in milk, such as choline, creatine, and carnitine [13]. Along with improving milk and component yields [14,15], supplementing rumen-protected forms of choline and methionine may also improve liver function and health [16,17]. In nonruminants, dietary choline and methionine are required to support hepatic fatty acid oxidation [18] while limiting oxidative stress and lipidosis [19]. Supplemental choline has limited liver lipid accumulation during the periparturient period [20] and feed restriction in dairy cows [21]. Dietary choline and methionine elicit lipotropic effects in nonruminants by supporting VLDL export and fatty acid oxidation [19,22]; however, their lipotropic action has not been elucidated in bovine and may be complicated by their use for methyl group donation. Methyl donor deficiency in nonruminants is also associated with hepatocellular injury and inflammation due to oxidative stress [18,22]. The process of FA oxidation produces reactive oxygen species (ROS) that are damaging unless they are neutralized by antioxidants. Oxidative stress occurs when the production of ROS exceeds antioxidant defense mechanisms present in the cell and begins damaging tissues [23,24].

We hypothesized that both choline and methionine can be a source of methyl groups for the transmethylation pathway and serve hepato-protective functions in bovine liver. The objective of this experiment was to examine the regulation of genes controlling methyl group transfer and lipid metabolism, and to quantify VLDL and reactive oxygen species secretion, in response to increasing concentrations of choline and methionine in the presence or absence of a fatty acid challenge. The use of an established in vitro model, cultures of primary neonatal bovine hepatocytes cultures, was selected in order to directly examine the role of choline and methionine, independent of whole-animal confounders.

## Materials and methods

### Isolation and cell culture

Primary bovine hepatocytes were isolated from Holstein bull calves <7 d of age by collagenase perfusion of the caudate process as previously described [25]. Isolated cells were plated on 35-mm Corning Primaria culture plates (Fisher Scientific, Hampton NH) in Dulbecco’s Modified Eagles Medium (DMEM 2902, 5.5 mmol/L glucose; Sigma-Aldrich, St. Louis, MO) supplemented with 20% fetal bovine serum (FBS) and 1% antibiotic, antimycotic solution (A5955, Sigma-Aldrich, St. Louis, MO). Four hours after initial plating, media was refreshed with a 10% FBS, 1% antibiotic, antimycotic DMEM media. All animal use and handling protocols were approved by the University of Wisconsin-Madison College of Agricultural and Life Sciences Animal Care and Use Committee.

### Treatments

Monolayer cultures of cells were maintained for 24 h and were approximately 80% confluent before exposure to treatment. Cells were randomly assigned to increasing concentrations of choline chloride (CC), DL-methionine (DLM), and a fatty acid cocktail in a 4×4×2 factorial arrangement. Although L-amino acids are known to be the active form for protein synthesis, a cell culture experiment confirmed that supplementation of primary neonatal bovine hepatocytes with methionine as D-Met, DL-Met, and L-Met does not result in differences in expression of transmethylation, gluconeogenic, oxidative, or inflammatory pathway genes, or abundance of ROS or glutathionine [26,27]. To control the concentration of methionine in treatment media, a methionine-free media was made according to the formulation for low glucose DMEM (2902; Sigma-Aldrich, St. Louis, MO) without adding methionine. The methionine-free treatment media was fortified with BME vitamins (6891; Sigma-Aldrich, St. Louis, MO) that contained 716 μmol/L CC and 227 μmol/L folic acid, and was completed to 1% bovine serum albumin (BSA; Merck Millipore, Billerica, MA) and 1% antibiotic, antimycotic solution. Sterile stock solutions of DLM were added to wells containing treatment media to achieve target concentrations of 16, 30, 100, and 300 μmol/L DLM. Because the fortified methionine-free DMEM contained 28 μmol/L CC via the BME vitamin solution, sterile stock solutions of CC were added to achieve treatments of 61, 128, 2028, and 4528 μmol/L CC. It is important to note that hepatocytes were cultured in a media that was not considered to be methyl donor deficient as folate was present and thus results may be attributed to the direct effects of choline and methionine as nutrients and not solely the addition of methyl carbon donors.

Treatments were replicated with and without addition of a fatty acid (FA) cocktail (1 mmol/L). Stocks of fatty acids C14:0, C16:0, C18:0, C18:1n-6 *cis*, C18:2n-6 *cis*, and C18:3n-3 *cis* (Sigma-Aldrich, St. Louis, MO) were bound to BSA by the method previously described [28] and combined in molar ratios to create a FA cocktail that mimicked the profile of circulating non-esterified fatty acids (NEFA) observed for dairy cows at the time of calving [29]. The FA cocktail was made up of 3% C14:0, 27% C16:0, 23% C18:0, 31% C18:1n-6 *cis*, 8% C18:2n-6 *cis*, and 8% C18:3n-3 *cis*. No additional BSA was added to treatment media supplemented with FA cocktail to maintain consistent concentration of BSA between FA and no FA treatments. To serve as a control for the analysis of ROS in the media, control cells were incubated in complete, low-glucose DMEM with and without FA that was not altered for CC or DLM. All reagents used were of cell-culture grade or the highest purity available (Sigma-Aldrich, St. Louis, MO). All treatments were applied in triplicate in independent preparations of cells from 4 Holstein calves.

### Quantifying ROS and VLDL content

Twenty-four hours after treatment exposure, media was harvested and triplicates pooled. An aliquot was used to quantify ROS immediately after harvest, while the remaining sample volume was stored at -80°C for later quantification of VLDL. Reactive oxygen species were quantified by fluorometric assay (Hydrogen Peroxide Cell-Based Assay, Cayman Chemical, Ann Arbor, MI). Concentration of ROS was normalized to the no FA treatment control within each cell preparation and expressed as the ratio of treatment with FA to treatment without FA.

Concentration of VLDL in treatment media of cells exposed to the FA cocktail was quantified using a bovine-specific ELISA technique (VLDL ELISA, NeoScientific, Cambridge, MA) designed for cell culture supernatants. The competitive immunoassay employed bovine-specific antibodies that bound to both apolipoprotein B100 and C in order to adhere VLDL particles to the plate before quantification by colorimetric assay. The assay that was available at the time of the experiment was validated using bovine serum and cell culture samples spiked with purified high-density and low-density lipoprotein standards that yielded a recovery of 102 ± 1%; however, although the ELISA kit is available under the same name, it is no longer made by the same manufacturer and has not been validated for this purpose. Data were normalized to the lowest CC and DLM treatment within each independent cell preparation and expressed as relative concentration of VLDL.

### RNA isolation and qPCR

After treatment media was collected, cells were rinsed with 1X PBS and harvested in 0.5 mL Trizol reagent (Invitrogen, Carlsbad, CA) and stored at -80°C until later RNA extraction and characterization. Total RNA was isolated from each plate using Trizol reagent and sample concentration was quantified and characterized using a spectrophotometer (Synergy H1, BioTek Instruments, Inc., Winooski, VT). A 100 μL pool containing balanced quantities of RNA from each triplicate was further purified using an on-column DNase I treatment and the RNeasy Mini Kit (Qiagen Inc., Thousand Oaks, CA). Cleaned RNA was re-quantified and characterized, with all samples having a ratio of absorbance at 260 nm and 280 nm equal to or greater than 1.9. A volume containing 0.5 μg of RNA was reverse transcribed in a 20 μL reaction using an iScript cDNA synthesis kit (Bio-Rad, Hercules, CA).

Abundance of mRNA was quantified using real-time quantitative PCR, SsoFast EvaGreen reagent (Bio-Rad, Hercules, CA), and the primers presented in Table 1. An equivalent quantity of cDNA from each sample was pooled and diluted to generate a standard curve. Water served as a no-template control and an RNA pool was included as a no reverse-transcription control for primer optimization. Transcripts were amplified as follows: 1 cycle at 95°C for 3 min and 45 cycles of 95°C for 5 sec and 55°C for 5 sec. Reaction efficiencies were between 90 and 110% based on standard curve analysis. All samples, standards, and controls were analyzed in triplicate and the quantification cycle (Cq) data were transformed using the standard curve method within Bio-Rad CFX Manager Software (version 3.1, Bio-Rad, Hercules, CA). The transcript abundance of potential reference genes was interrogated to ensure the absence of a treatment effect. Data for genes of interest were then normalized to the corresponding mean transcript abundance of two reference genes, 18S ribosomal RNA (*18S*) and ribosomal protein S9 (*RPS9*), and are expressed as arbitrary units of mRNA abundance.

**Table 1:** Primer sequence used for quantitative real-time PCR.

Gene of interest	GenBank accession	Position	Sequence
*18S*	NR_036642.1	Forward	5’-ACCCATTCGAACGTCTGCCCTATT-3’
		Reverse	5’-TCCTTGGATGTGGTAGCCGTTTCT-3’
*RPS9*	NM_001101152.2	Forward	5’-CCTCGACCAAGAGCTGAAG-3’
		Reverse	5’-CCTCCAGACCTCACGTTTGTTC-3’
*BHMT*	NM_001011679.1	Forward	5’-TGGGGGCAAAAATGTCAAGAAG-3’
		Reverse	5’-TCCGTCTCCGATGATGACCT-3’
*MTR*	NM_001030298.1	Forward	5’-GCCCCTGAAAGGCAACAATG-3’
		Reverse	5’-CATCCGGTAGGCCAAGTGTT-3’
*MTHFR*	NM_001011685.1	Forward	5’-TGAGGGGAGACCCCATAGGT-3’
		Reverse	5’-TCAGGTGCTTCAGATCAGCC-3’
*MAT1A*	NM_001046497.1	Forward	5’-GCCTGTGAGACGGTGTGTAA-3’
		Reverse	5’-TAGCCAAACATCAGCCCCTG-3’
*PEMT*	NM_182989.3	Forward	5’-GCTGTCCTTGCCATTGTCTTC-3’
		Reverse	5’-TCGGGTCTTGTGTTCCCATC-3’
*MTTP*	NM_001101834.1	Forward	5’-ACCTGTGCTCCTTCATCTAATTCAT-3’
		Reverse	5’-GCTAGCCAGGCCTCTCTTGA-3’
*CPT1A*	NM_001304989.1	Forward	5’-TTCGCGATGGACTTGCTGTA-3’
		Reverse	5’-TTTCCTCCCGGTCCAGTTTG-3’

### Statistical analysis

Data were analyzed for treatment effects using the MIXED procedure of SAS 9.4 (SAS Institute Inc., Cary, NC). The model accounted for the fixed effect of treatment, all two-way and three-way interactions, and the random effect of cell preparation. Non-symmetrical linear and quadratic contrast statements were applied to test the effect of increasing concentration of CC and DLM. Linear effects were considered significant when *P* ≤ 0.05 and a trend toward significance when 0.05 < *P* ≤ 0.10. Data are reported as least-square mean and standard error of the mean.

## Results

**Fig 2:**
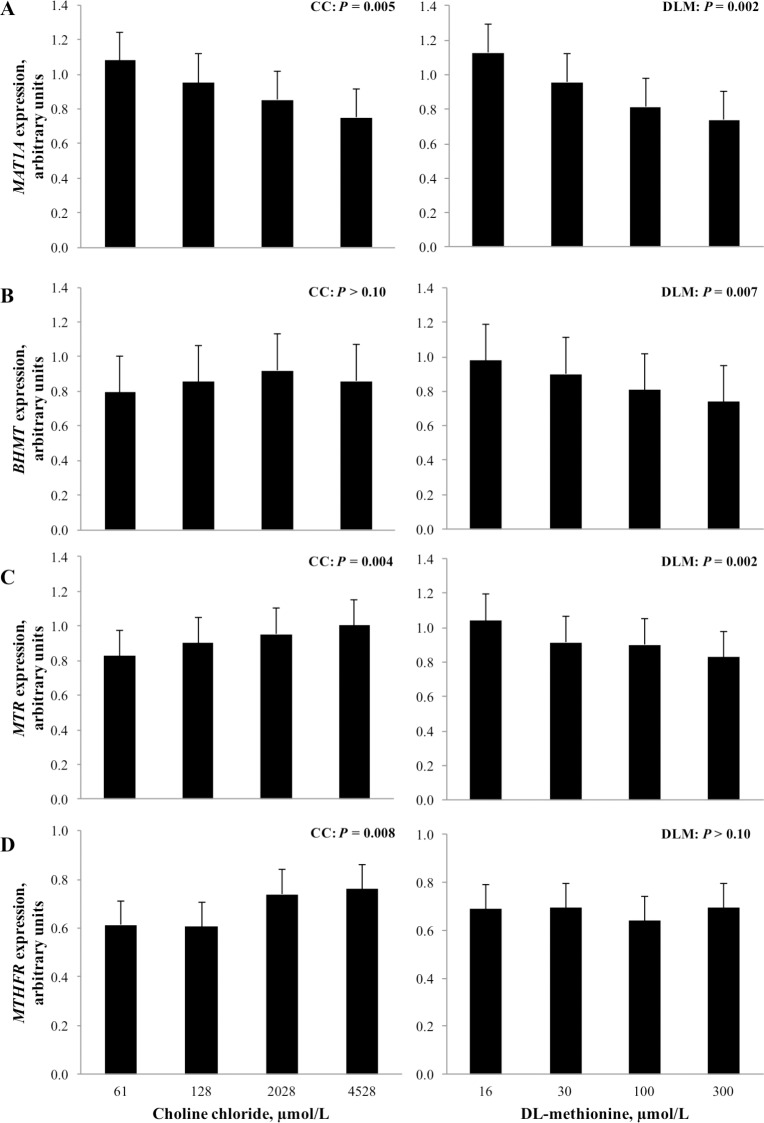
Expression of genes related to choline and methionine metabolism in the transmethylation pathway in neonatal bovine hepatocytes exposed to increasing concentrations of choline chloride (CC) and DL-methionine (DLM). The *P*-values for linear effects of CC and DLM are shown; there were no interactions between CC and DLM. Values are least squares means, with SE represented by vertical bars. Methionine adenosyltransferase 1A (*MAT1A*), betaine-homocysteine S-methyltranferase (*BHMT*), 5-methyltetrahydrofolate-homocysteine methyltransferase (*MTR*), methylenetetrahydrofolate reductase (*MTHFR*).

**Fig 3:**
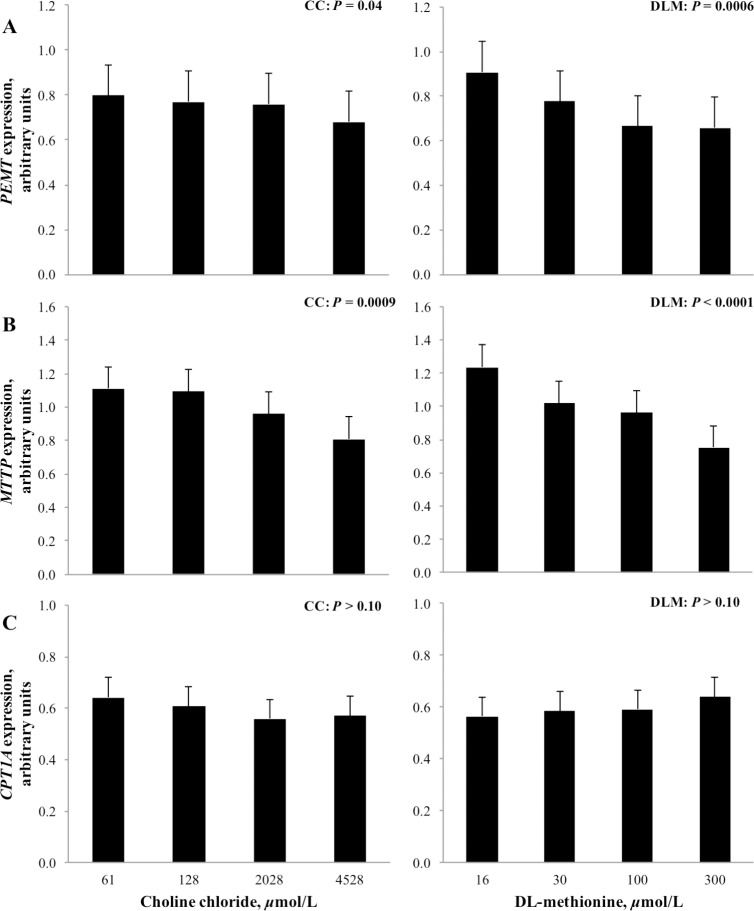
Expression of genes related to lipid metabolism and oxidation in neonatal bovine hepatocytes exposed to increasing concentrations of choline chloride (CC) and DL-methionine (DLM); there were no interactions between CC and DLM. Values are least squares means, with SE represented by vertical bars. Phosphatidylethanolamine N-methyltransferase (*PEMT*), microsomal triglyceride transfer protein (*MTTP*), carnitine palmitoyltransferase 1A (*CPT1A*).

**Fig 4:**
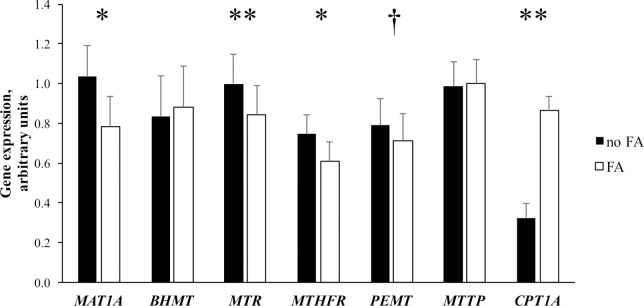
Expression of genes related to pathways of metabolism in transmethylation, lipid metabolism, and lipid oxidation in neonatal bovine hepatocytes exposed to increasing concentrations of choline chloride and DL-methionine in the presence (closed bars) or absence (open bars) of a 1 mmol/L fatty acid cocktail. The main effects of fatty acid (FA) treatment are shown; there were no interactions between the methyl donor or FA treatment. Values are least squares means, with SE represented by vertical bars. Effects are denoted as tendency (P ≤ 0.10, †) and significant (*P* ≤ 0.05, *; *P* ≤ 0.001, **). Methionine adenosyltransferase 1A (*MAT1A*), betaine-homocysteine S-methyltranferase (*BHMT*), 5-methyltetrahydrofolate-homocysteine methyltransferase (*MTR*), methylenetetrahydrofolate reductase (*MTHFR*), phosphatidylethanolamine N-methyltransferase (*PEMT*), microsomal triglyceride transfer protein (*MTTP*), carnitine palmitoyltransferase 1A (*CPT1A*).

No interactions were detected between CC, DLM and FA treatment for any of the genes examined, therefore the 2- and 3-way interactions are not shown or discussed. Because quadratic effects were not significant, only linear effects of CC and DLM treatment for genes associated with methyl carbon metabolism are presented in Figs 2 & 3. Fatty acid treatment differentially affected the genes examined and the main effect of FA is shown in Fig 4.

Increasing the concentration of CC and DLM in media linearly decreased (*P* < 0.05) expression of methionine adenosyltransferase 1A (*MAT1A*, EC 2.5.1.6). Increasing concentration of DLM in media linearly decreased (*P* < 0.05) expression of betaine-homocysteine S-methyltranferase (*BHMT*, EC 2.1.1.5), while CC and exposure to a 1 mmol/L FA cocktail did not alter (*P* > 0.10) expression of the gene (Figs 2 and 4). Expression of 5-methyltetrahydrofolate-homocysteine methyltransferase (*MTR*, EC 2.1.1.13) linearly increased (*P* < 0.05) as CC concentration increased and decreased (*P* < 0.01) linearly with increased DLM. Expression of methylenetetrahydrofolate reductase (*MTHFR*, EC 1.5.1.20) was linearly increased (*P* < 0.05) as CC increased in the media. Concentration of DLM did not alter (*P* > 0.10) *MTHFR* expression. Treatment with FA decreased (*P* < 0.01) the expression of *MTR*, *MTHFR*, and *MAT1A* (Fig 4).

Linear effects of treatment on the expression of phosphatidylethanolamine N-methyltransferase (*PEMT*, 2.1.1.17), carnitine palmitoyltransferase 1A (*CPT1A*, EC 2.3.1.21), and microsomal triglyceride transfer protein (*MTTP)* are shown in Fig 3. Increasing concentration of CC and DLM linearly decreased (*P* < 0.05) the expression of both *PEMT* and *MTTP*. While the exposure of FA tended to decrease (*P* = 0.07) the expression of *PEMT*, it did not alter (*P* > 0.10) *MTTP* expression (Fig 4). Neither CC or DLM altered (*P* > 0.10) *CPT1A* expression; however, it was significantly induced (*P* < 0.01) by FA treatment.

**Fig 5:**
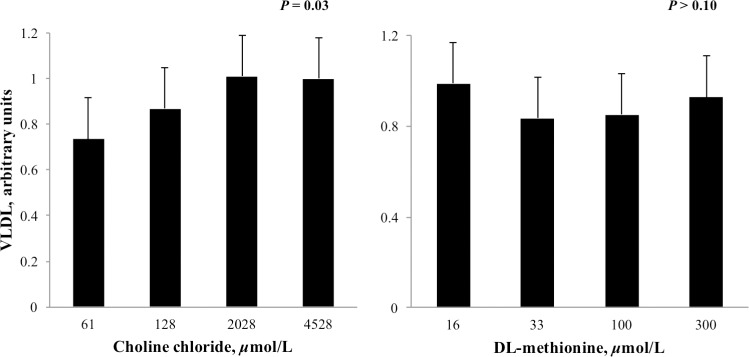
Relative concentration of VLDL in cell culture media that incubated neonatal hepatocytes treated with increasing concentrations of choline chloride (CC) and DL-methionine (DLM) and a 1 mmol/L fatty acid cocktail for 24 h. Concentration of VLDL was normalized to the lowest CC and DLM treatment within cell preparation. Increasing CC linearly increased (*P* = 0.03) VLDL release from neonatal hepatocytes and treatment with DLM had no effect (*P* ≥ 0.10); there was no interaction between CC and DLM.

**Fig 6:**
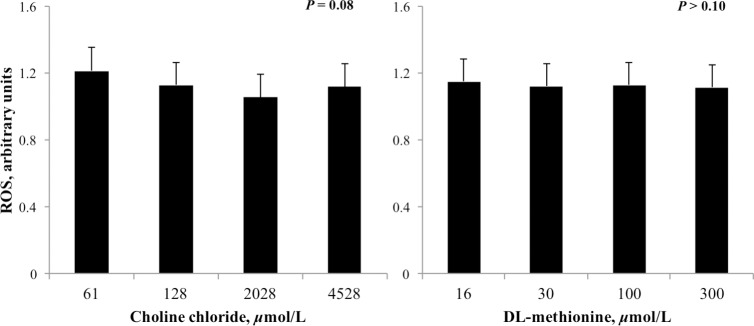
Relative concentration of reactive oxygen species (ROS) in media that incubated neonatal hepatocytes treated with increasing concentrations of choline chloride (CC) and DL-methionine (DLM) and a 1 mmol/L fatty acid cocktail for 24 h. Concentration was normalized to controls and expressed as the ratio of treatment with FA to treatment without FA. Increasing CC tended to linearly decrease (*P* = 0.08) the accumulation of ROS and DLM had no effect (*P* ≥ 0.10); there was no interaction between CC and DLM.

The concentration of VLDL in media was linearly increased (*P* < 0.05) by increasing CC but was unaffected by DLM (Fig 5). Increasing the concentration of CC, but not DLM, tended to decrease (*P* = 0.08) the accumulation of ROS in cell culture media (Fig 6).

## Discussion

Utilization of choline and methionine as nutrients to support the anabolic output of production in transition dairy cows may be confounded by requirements to fulfill biological roles to support maintenance metabolism, such as methyl donation and protein synthesis, prior to fulfilling the demands associated with the anabolic output of growth and production; however, these confounding factors are difficult to elucidate in vivo. For this reason, a well-established primary bovine hepatocyte in vitro model using hepatocytes isolated from neonatal [25,30–32] or weaned eight or ten week old calves [33,34] was used in the current work to directly examine the role of methionine and choline on hepatocyte metabolism. Primary bovine hepatocytes in the current research were derived from neonatal calves and the hepatocytes were subsequently incubated in media that reflects the long-chain fatty acid composition found in the plasma of ruminating animals, in order to examine the effect of fatty acids, choline, and methionine on methyl group metabolism and ROS responses. Treatment concentrations were selected guided by information available regarding bovine arterial concentrations; however, it is important to note that nutrient concentrations can be 50% greater in the portal vein than in arteries [35]. Plasma concentrations of methionine have been consistently reported between 15 and 25 μmol/L in lactating cows [5,36–38], but range from 30 to 40 μ*M* [5,37,39] and can increase up to 144 μmol/L [38] when supplemental rumen-protected methionine is fed. Circulating choline is present in many different forms including free choline, phosphocholine, PC, lysophosphatidylcholine, glycerophosphocholine, and direct products including betaine and sphingomyelin; most of which can be used to supply choline or choline-derived methyl groups to the liver [3]. Uptake kinetics and metabolism of choline in perfused rat liver and isolated rat hepatocytes revealed that the cells were responsive to concentrations that ranged from 20 to 4500 μmol/L [40,41]. Recent characterization of plasma and milk choline metabolites in dairy cattle during different stages of lactation demonstrated that total plasma choline metabolite concentrations range from 1,305 to 14,241 μmol/L [42]. Reflectively, at 4×4×2 factorial design of 16, 30, 100, 300 μmol/L DLM and 61, 128, 2028, and 4529 μmol/L CC with or without the addition of the 1 mmol/L fatty acid cocktail was used. The linear responses of gene expression to these CC and DLM doses did not appear to plateau within the range of concentrations examined here.

### Methyl carbon metabolism

The overlapping methyl donating properties of choline and methionine are integrated in the transmethylation cycle where pathways of their catabolism interact (Fig 1). Exogenously supplied or endogenously regenerated methionine can serve as a methyl donor through the generation of SAM, via the enzyme activity of MAT1A. Decreased *MAT1A* expression with CC and DLM treatment in the current experiment is not likely indicative of decreased SAM synthesis or a diminished need for SAM methylation, instead methionine-stimulated SAM synthesis may have increased methylation of the *MAT1A* promoter region to decrease gene expression and maintain stable SAM concentrations, as previously shown [43]. During states of increased demand for SAM and methylated compounds, such as during lactation, rather than the absolute rate of SAM synthesis changing, rates of dispensable methylation are decreased [13]. Methylation pathways that form non-anabolic byproducts, such as sarcosine, can be altered to maintain SAM concentrations [6,13,44] and resupply methyl groups to the folate methyl pool ([45]; Fig 1), constituting important mechanisms for maintaining constant cellular SAM concentrations [46]. In the present study, *MAT1A* expression was also decreased by FA treatment. If hepatocytes responded to the FA challenge by decreasing rates of dispensable methylation to support FA metabolism, SAM synthesis could have been reciprocally downregulated to prevent increases in SAM that could disturb functional methyltransferases, possibly explaining the observed decrease in *MAT1A* expression. There was no effect of supplementing rumen-protected methionine on hepatic *MAT1A* expression during a transition cow study, nor was *MAT1A* expression altered from -10 d prepartum to 21 d postpartum when circulating NEFA concentrations were greater than 1.2 mmol/L [47]. These differences could reflect responsiveness of *MAT1A* expression to other changes associated with the transition to lactation period in vivo, or could indicate differences due to the in vitro system.

The enzyme BHMT uses methyl groups donated from betaine, an intermediate of choline catabolism, to remethylate homocysteine (Fig 1) [48]. The lack of response of bovine *BHMT* to CC in the current work may be attributed to a comparatively low enzyme activity of BHMT in other ruminants [1]. Although nonruminants rely largely on the activity of BHMT to regenerate methionine [1,49], the activity of BHMT in sheep liver and pancreas was more than 5 times lower than in rats [1]. Incubation of neonatal bovine hepatocytes with increasing concentrations of DLM linearly decreased *BHMT* expression which has been similarly observed with DLM, and other forms of methionine [26,50]. It is not unexpected that increasing methionine decreased the expression of *BHMT*, given that supplemental methionine appeared to decrease methionine regeneration in sheep and growing steers [51,52].

While BHMT activity is comparatively lower in fully functional ruminants compared to nonruminants, MTR activity is comparatively higher and primarily responsible for methionine regeneration in other ruminant species [1], and connects the supply of methyl groups carried in the folate pathway to the regeneration of methionine and SAM synthesis. Similarly, increasing methionine decreased *MTR* expression in growing steers [52]. Although supplemental methionine increased *MTR* mRNA abundance in prepartum cows [47], it had no effect postpartum [53,54] or later in lactation [54]. The methionine-stimulated decrease in *MTR* expression observed in this and a similar experiment [50] and may reflect a decreased need for methionine regeneration as more methionine was supplied.

Coordinated increases in *MTR* and *MTHFR* expression with CC treatment suggests that choline can support methionine regeneration and may possibly prevent the catabolism of exogenous methionine in the transmethylation pathway. The current data provides evidence of a direct mechanism of choline to spare methionine within bovine hepatocytes. Consistent with this, infusion of choline decreased the irreversible loss of methionine by 18 to 25% in sheep [10], possibly sparing it from catabolism for use in protein synthesis. Preferential use of exogenous methionine versus endogenously synthesized, either by transmethylation or conversion of methionine analogues or enantiomers, has been demonstrated in both human and mouse cell culture [55] and in splanchnic metabolism research in dairy cows [35,56]. Taken together, providing alternative methyl donors to the transmethylation pathway and preventing catabolism of preformed methionine may be beneficial to maintain hepatic proteomic function.

The addition of NEFA to the media did not alter the expression of *BHMT*, but decreased *MTR* and *MTHFR* mRNA abundance, regardless of CC and DLM concentration. This apparent decrease in methionine regeneration may have been mediated by decreases in dispensable methylation, as discussed above, or changes in transmethylation flux. Alternatively, NEFA may have mediated a shift between these pathways of methyl donation. Around the time of calving, *BHMT* was upregulated and *MTR* downregulated in cows experiencing NEFA mobilization and hepatic lipidosis [47,57]. This coordinated shift suggests that dairy cows may rely on *BHMT* and sources of betaine for methionine regeneration more during the transition period, potentially allowing for regulation in response to changes associated with the transition to lactation. If so, supplying betaine or choline may be even more important during the transition to lactation period to help support the regeneration of methionine and prevent the catabolism of membrane PC.

### Lipid oxidation and transport

While choline can supply methyl groups, the nutrient is required for the synthesis of PC, an integral phospholipid for VLDL synthesis [11], lipid membrane integrity [12] and bile secretions [58]. In addition to generation of PC from choline via the CDP-pathway, PC can be synthesized by successive methylation of phosphatidylethanolamine (PE) by PEMT. Isotopic labeling of choline supplied to rat hepatocyte cultures revealed that PC synthesized from choline via the CDP-pathway, but not the PEMT pathway, was preferentially found in lipoprotein secretions (specifically VLDL fractions) [11]. Although both biochemical pathways of PC generation are possible, this and other evidence of discriminatory PC use based on source [59] supports the role of choline-activated PC in VLDL export. The comparatively low rate of hepatic VLDL synthesis and export by bovine and other ruminant species is well documented [60,61], and may be attributed to diminished PC synthesis, limited by availability of choline for CDP-pathway activation [62]. Consistent with this, increasing CC in the current work increased VLDL secretion, likely through increases in CDP-pathway generated PC, given that *PEMT* expression was downregulated by both CC and DLM.

Expression of *MTTP* has been used previously as an indicator of hepatic VLDL synthesis in dairy cows [63,64]; however, expression of *MTTP* may not limit VLDL export. The uncoordinated decrease in *MTTP* expression but increased VLDL export with increasing CC (Fig 5) suggests that *MTTP* did not induce or limit VLDL export in this study. Previously, feed restriction to induce hepatic lipidosis did not alter hepatic MTTP protein activity in vivo [65]. Similarly, *MTTP* expression was not changed during incubation of cultured neonatal bovine hepatocytes with up to 2.0 mmol/L NEFA, even though intracellular TG accumulation increased exponentially [65]. Taken together, bovine *MTTP* does not seem to be a reliable indicator of VLDL secretion in vivo or in vitro.

Expectedly, *CPT1A* mRNA abundance was significantly increased by FA treatment. Gene expression of *CPT1A* is regulated by long chain FA, and their presence markedly increased mRNA abundance of *CPT1A* in cultured rat hepatocytes [66]. Additionally, in neonatal bovine hepatocytes, *CPT1A* mRNA and CPT1A protein abundance were strongly correlated [67]. Conversely, neither CC or DLM affected the expression of *CPT1A* in this experiment. Similarly, supplementation of rumen-protected choline [63] or methionine [64] did not affect in vivo expression of *CPT1A* in peripartum cows. Previously it was speculated that the lipotropic effects of choline [20,63] could be attributed to a choline stimulated increase in FA oxidation [21]. The lack of CC effect on *CPT1A* suggests this is not mediated by increasing FA supply for oxidation, or that *CPT1A* was not limiting in this experiment; however, this does not preclude the potential for choline and methionine to support FA oxidation by other direct or indirect means.

In the present study, supplementation with DLM did not alter ROS; however, the addition of choline tended to decrease ROS accumulation (Fig 6). Although greater concentrations of glutathione in the liver of methionine supplemented dairy cows [57], growing steers [52], and neonatal bovine hepatocytes [26] have been reported, ROS either did not change [26] or was not examined. Given that VLDL export was not increased with DLM, catabolism of those FA not exported could have produced additional ROS which may have negated any methionine-mediated reduction in ROS. Despite being a product of metabolism, ROS accumulation and subsequent oxidative stress can present a challenge to dairy cattle [68], although the balance and optimal concentrations of ROS and glutathione are not fully understood in bovine hepatocytes or dairy cattle. The mechanism of CC to limit ROS accumulation in bovine hepatocytes remains to be elucidated but may reflect increased export of FA from the cell.

## Conclusions

While both choline and methionine supplementation influence production and growth of dairy cattle, the lack of interaction in the present study supports preferential uses for choline and methionine in bovine hepatocytes. Increasing supply of choline appeared to support methionine regeneration from the folate methyl pool, while methionine supply decreased the need for homocysteine remethylation. This offers evidence that choline could support the transmethylation pathway, sparing methionine from catabolism for its preferential use in protein synthesis and later anabolic output. In addition, choline uniquely supported hepatic lipid metabolism by increasing VLDL export and limiting the accumulation of ROS. The data discussed here supports that the mechanism of choline to decrease hepatic TG is by increasing the synthesis and export of VLDL.

## Supporting information

S1 Data Data table. (XLSX)
